# A Dynamic Tensile Method Using a Modified M-Typed Specimen Loaded by Split Hopkinson Pressure Bar

**DOI:** 10.3390/ma18010149

**Published:** 2025-01-02

**Authors:** Yuan Lin, Jitang Fan, Xinlu Yu, Yingqian Fu, Gangyi Zhou, Xu Wang, Xinlong Dong

**Affiliations:** 1 Key Laboratory of Impact and Safety Engineering (Ningbo University), Ministry of Education, Ningbo 315211, China; 2111081013@nbu.edu.cn (Y.L.); wangxu@nbu.edu.cn (X.W.); 2 Faculty of Mechanical Engineering and Mechanics, Ningbo University, Ningbo 315211, China; zhougangyi@nbu.edu.cn; 3 State Key Laboratory of Explosion Science and Technology, Beijing Institute of Technology, Beijing 100081, China; jitang_fan@hotmail.com; 4 College of Science and Technology, Ningbo University, Ningbo 315300, China; yuxinlu@nbu.edu.cn

**Keywords:** dynamic tensile, split Hopkinson pressure bar, M-type specimen, strain rate effect, 3D-printed stainless steel

## Abstract

Obtaining reliable dynamic mechanical properties through experiments is essential for developing and validating constitutive models in material selection and structural design. This study introduces a dynamic tensile method using a modified M-type specimen loaded by a split Hopkinson pressure bar (SHPB). A closed M-type specimen was thus employed. Finite element simulations and experiments were used to validate the design of the M-type specimen, which was fabricated using 17-4PH (precipitation hardening) stainless steel powder with a 3D (three-dimensional) selected laser melting (SLM) printer. After verifying force balance and uniform deformation in the tensile region, tensile tests were conducted across strain rates from quasi-static to a strain rate of 5900 s^−1^. The results demonstrated that this method effectively assessed the dynamic tensile behaviors of stainless steel at high strain rates, and achieved both ultra-high strain rates and large plastic deformation.

## 1. Introduction

In designing and analyzing engineering structures subjected to impact loads, it is crucial to obtain reliable dynamic mechanical properties of materials through experiments [1,2,3]. These properties are essential for developing and validating dynamic constitutive models of engineering materials [4,5,6].

Previously, various methods have been explored for measurement of the mechanical response of materials at high strain rates [7,8,9,10,11]. For strain rates in the range of ≤10 s^−1^, ordinary hydraulic servo-controlled testing machines have been used to load and test the quasi-static and low-speed tensile behaviors of most engineering materials [12,13]. For higher strain rates in the range of 10 $\dot{<r<}$ 10^3^ s^−1^, an intermediate strain rate, many special devices have been developed, including high-speed servo-hydraulic testing machines, hybrid testing apparatus, the drop tower, and the flywheel device [7,14,15,16,17,18]. For high strain rate testing > 10^3^ s^−1^, the split Hopkinson tensile bar (SHTB), flyer plate impact test and expansion-ring techniques have been used to characterize the dynamic response of materials under impact loading [1,4,8,19,20,21,22,23,24,25]. The Hopkinson bar technique, known for its simplicity and high accuracy, is widely used to study the dynamic compressive/tensile properties of materials at high strain rates. The conventional Hopkinson bar technique consists of a striker bar, an incident bar, and a transmission bar, with the specimen sandwiched between the incident and transmission bars.

However, testing dynamic tensile properties at high strain rates present significant challenges. Over recent decades, various split Hopkinson tensile bar systems have been developed to address these challenges, particularly focusing on how the specimen is connected to the incident and transmission bars [22,23,24]. Common connection methods include bonding, threading, or pinning the specimen to the bars [25,26,27]. Bonding techniques often lack the strength needed for testing higher-strength materials, while bolt and pin connections require extremely high machining and assembly precision to ensure accurate results.

Nicholas [1] emphasized that for threaded specimens, achieving a precise, gap-free fit between the specimen and the bar is essential for accurate high-strain-rate tensile testing. Even minor gaps or misalignments can result in reflected stress waves, which are amplified and introduce significant errors into the data.

The most widely used approach in Hopkinson bar setups involves screwing the tensile specimen with threaded shoulders into the rod ends. However, under tensile loading, separation between the threaded shoulder and the rod end creates a free surface that reflects stress waves, causing inaccuracies.

Bonding with organic adhesives eliminates gaps associated with threaded or pinned connections but introduces new challenges. The high viscoelasticity of adhesives can lead to large deformations, distorting strain calculations based on one-dimensional stress wave theory [27,28]. Additionally, the long curing times of adhesives reduce experimental efficiency.

Mohr and Gary [29] addressed the challenges associated with Hopkinson tensile bar testing by proposing the use of a Hopkinson compression bar to load M-type specimens. This approach transforms compressive stress waves into dynamic tensile loads, eliminating the connection issues between the specimen and the rod. However, since this method was proposed, it has not been widely adopted or experimentally validated, possibly due to the challenges in manufacturing M-type specimens. Additionally, thin sheet materials, such as automotive steel sheets, which often require dynamic tensile testing, are unsuitable for M-type specimen fabrication.

Recently, with the growing use of additive manufacturing, the dynamic tensile behavior and failure characteristics of 3D-printed metal materials have gained significant attention [30,31,32,33,34,35,36,37,38,39]. This has highlighted the need to develop dynamic uniaxial tensile testing methods for small specimens. In response, we conducted experiments and finite element analysis on dynamic tensile tests using M-type specimens. Our study focused on improving the design of the specimen shape from open-M to close-M. Then, critical factors were examined to assess their effects on the mechanical response of 3D-printed metal materials, such as the validity of the one-dimensional stress assumption, the uniformity of Hopkinson experiments, data processing techniques, loading waveforms, and elastic corrections in closed M-type specimens. Based on these pre-works, a series of dynamic tests was performed with different strain rates.

This research aimed to provide a dynamic tensile experimental method to determine the reliable mechanical properties of 3D-printed stainless steel using a modified close M-type specimen. The results will to be helpful in developing and validating constitutive models of materials with high strain-rates.

## 2. Experimental Methodology

### 2.1. 3D-Printed Material

**Figure 1:**
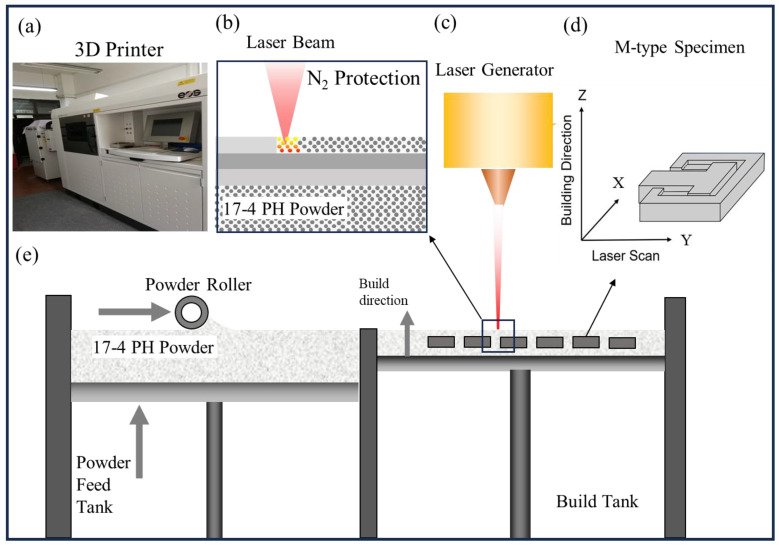
Schematic diagram of the 3D-printed building process: (**a**) EOS M280 3D printer, (**b**) schematic diagram of laser scanning, (**c**) laser beam, (**d**) M-type specimen, and (**e**) build process of specimen.

The M-type tensile specimens were produced using a SLM facility (EOS GmbH, EOS M280, that was equipped with a 400 W Gaussian beam fiber laser with a focal laser beam diameter of 80 μm), as shown in Figure 1. Powder of 17–4 PH stainless steel, with a particle size range of 10 to 80 μm, was used for fabrication. The powder’s chemical composition was: 74.54% iron (Fe), 0.61% manganese (Mn), 2.69% copper (Cu), 0.63% silicon (Si), 0.05% carbon (C), 17.54% chromium (Cr), and 4.36% nickel (Ni). The process parameters were optimized as follows: laser power of 400 W, laser scanning speed of 1000 mm/s, layer thickness of 20 μm, and preheated temperature of 80 °C. Nitrogen shielding was applied during the printing process to prevent oxidation. To ensure consistent performance, 16 M-type specimens were printed in a single batch to ensure consistent performance across all samples. The parts were cut from the substrate using wire electrical discharge machining after production.

### 2.2. M-Type Tensile Method

#### 2.2.1. Basic Framework

**Figure 2:**
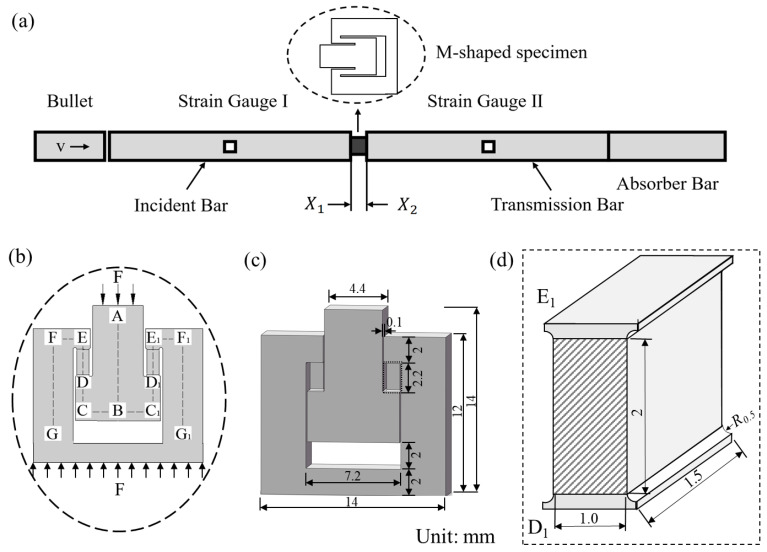
The loading mechanism of the M-type specimen: (**a**) SHPB loading devices, (**b**) tensile gauge of DE and D_1_E_1_ in M-type specimen in (**a**), (**c**) detail of specimen, and (**d**) tensile gauge section of D_1_E_1_ shown in the dash box of (**c**).

The loading mechanism of the M-type specimen is shown in Figure 2. The M-type specimen sandwiched between the incident bar and transmission bar in the SHPB device is shown in Figure 2a. This method converts the compressive load at two end surfaces of specimen into tensile forces on gauge sections of DE and D_1_E_1_, as shown in Figure 2b. By measuring the load and deformation in these sections, the material’s tensile properties can be estimated. The detailed dimensions of M-type specimen are shown in Figure 2c. This method works for both quasi-static and dynamic tensile tests, and can easily be conducted using a Hopkinson pressure bar.

This experimental approach simplifies installation and minimizes errors associated with reflected stress waves from connection gaps. To enhance the overall stiffness of the specimen, the thickness of sample was increased to be 3.5 mm. The thickness of the tensile gauge sections, DE and D_1_E_1_, was designed to be 1.5 mm, as shown in Figure 2d. Due to their width being significantly greater than their thickness, these sections could be treated as operating under one-dimensional tensile strain.

#### 2.2.2. SHPB

The SHPB apparatus was used to evaluate the high-rate behavior of materials, as shown in Figure 2a. In this setup, the specimen was sandwiched between the incident bar and transmission bar. A gas gun launched a bullet at a predetermined velocity, impacting the free end of the incident bar. This impact generated a compressive stress wave, which propagated through the incident bar and applied a dynamic compressive load to the specimen.

For a conventional cylindrical specimen, a portion of the stress wave was transmitted to the transmission bar, while the remainder was reflected back into the incident bar. Strain gauges affixed to both the incident and transmission bars measured and recorded the incident wave, *ε_i_*(*t*), the reflected wave, *ε_r_*(*t*), and the transmitted wave, *ε_t_*(*t*). Based on these measurements, and through the application of one-dimensional stress wave analysis, the strain rate, strain, and stress experienced by the specimen were subsequently calculated as follows:

(1) $$\dot{\varepsilon }\left(t\right)=-2\frac{C_{0}}{L_{s}}\varepsilon_{r}\left(t\right)$$

(2) $$\varepsilon (t)=-2\frac{C_{0}}{L_{s}}\int_{0}^{t}\varepsilon_{r}(t)dt$$

(3) $$\sigma \left(t\right)=\frac{A_{b}E}{A_{s}}\varepsilon_{t}\left(t\right)$$

where, *E* represents the elastic modulus of the SHPB material (steel), *E* = 210 GPa. *A_b_* refers to the bar’s cross-sectional area, while *C*_0_ indicates the elastic wave speed within the bar, *C*_0_ = 5188 m/s. The cross-sectional area of the cylindrical specimen is denoted by *As*, and *Ls* stands for the specimen’s length.

In this study, the incident and transmission bars both had a diameter of 14.5 mm, thus *A_b_* = 165 mm^2^. By varying the bullet’s velocity and length (150 mm, 200 mm, and 300 mm), stress pulses with different amplitudes and wavelengths were generated. These variations allowed us to apply different strain rates to the specimen.

#### 2.2.3. Stress and Strain

In the SHPB experiment with uniform cylindrical specimens, stress and strain were derived using Equations (1)–(3). However, for the M-type specimen with a non-uniform cross-section, stress and strain had first to be converted into the forces at the specimen’s ends and the total deformation using Equations (4) and (5). This step allowed the calculation of stress and strain in the tensile section.

(4) $$U\left(t\right)=-2C_{0}\int_{0}^{t}\varepsilon_{t}\left(t\right)dt$$

(5) $$F\left(t\right)=A_{b}E\varepsilon_{t}\left(t\right)$$

When calculating the tensile stress, it is assumed that equal forces are applied to both ends of the specimen, following the stress equilibrium condition expressed as follows:

(6) $$\varepsilon_{i}\left(t\right)+\varepsilon_{r}\left(t\right)=\varepsilon_{t}\left(t\right)$$

For strain calculations, the measured displacement *U* at the specimen ends includes both the deformation of the tensile section and the elastic deformation of other parts. To isolate the tensile section’s displacement, the elastic deformation of the other sections must be excluded. If the displacement caused by plastic deformation in the tensile section ED is *U_p_*, and the elastic deformation displacement in other parts is *U_e_*, the relationship is defined by the following equation:

(7) $$U_{P}(t)=U(t)-U_{e}(t)$$

It is assumed that *U_e_* is related to the overall stiffness of the specimen and is proportional to the applied load force *F*(*t*), which is the compressive force at the ends of the specimen:

(8) $$F\left(t\right)=KU_{e}\left(t\right)$$

where *K* represents the stiffness coefficient of the specimen which was obtained using the numerical simulation described in Section 3.4. The engineering plastic strain in the tensile section is expressed as follows:

(9) $$\varepsilon_{yy}^{p}(t)=\frac{U_{p}(t)}{l_{0}}=\frac{U(t)-U_{e}(t)}{l_{0}}=\frac{U(t)-F(t)/K}{l_{0}}$$

where *l*_0_ is the initial length of the tensile section. The engineering stress in the tensile section is determined as follows:

(10) $$\sigma_{yy}^{l}\left(t\right)=\frac{F\left(t\right)}{A_{s}}$$

where *A_s_* = 2*W*_0_·*H*_0_ is the cross-sectional area of the tensile section, with *W*_0_ as the width and *H*_0_ as the thickness of the tensile section. Accordingly, the true stress and true strain are as follows:

(11) $$\sigma_{yy}^{t}\left(t\right)=\frac{F\left(t\right)}{A_{s}}\left(1+\varepsilon_{yy}^{p}\left(t\right)\right)$$

(12) $$\varepsilon_{yy}^{t}(t)=\ln(1+\varepsilon_{\mathrm{yy}}^{p}(t))$$

Thus, by measuring the load *F*(*t*) and displacement *U*(*t*) at the ends of the M-shaped specimen and determining the stiffness coefficient *K*, the stress–strain curve of the tensile section can be obtained and analyzed.

Furthermore, using Equations (6)–(12), the dynamic tensile stress $\sigma_{yy}^{t}\left(t\right)$, the plastic strain $\varepsilon_{yy}^{t}\left(t\right)$, and the corresponding strain rate $\dot{\varepsilon }\left(t\right)$ in the SHPB test can be calculated as follows:

(13) $$\sigma_{yy}^{t}=\frac{EA_{b}\varepsilon_{t}(t)}{A}(1+\frac{2C_{0}\int_{0}^{t}\left[\varepsilon_{i}(t)-\varepsilon_{t}(t)\right]dt}{l_{0}}-\frac{EA_{b}\varepsilon_{t}(t)}{l_{0}K})$$

(14) $$\varepsilon_{yy}^{t}=\ln(1+\frac{2C_{0}\int_{0}^{t}\left[\varepsilon_{i}(t)-\varepsilon_{t}(t)\right]dt}{l_{0}}-\frac{EA_{b}\varepsilon_{t}(t)}{l_{0}K})$$

(15) $$\dot{\varepsilon }\left(t\right)=-\frac{2C_{0}}{l_{0}}\left[\varepsilon_{i}\left(t\right)-\varepsilon_{t}\left(t\right)\right]+\frac{EA_{b}\varepsilon_{t}^{’}\left(t\right)}{l_{0}K}$$

### 2.3. Experimental Program

**Figure 3:**
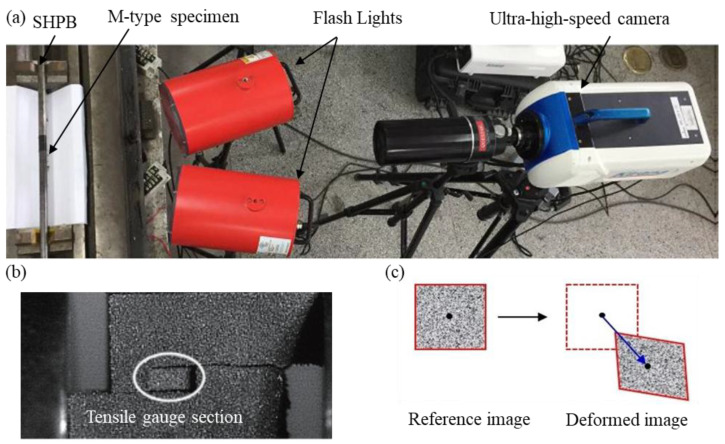
The experimental loading and measuring setups: (**a**) experimental apparatus of SHPB, ultra-high-speed camera, and flash lights, (**b**) the image captured by camera, and (**c**) the principle of DIC method.

Quasi-static and dynamic tensile tests were conducted on 3D-printed stainless steel M-type specimens. There was one quasi-static tests at the strain rate of 0.002 s^−1^ and three dynamic tests with strain rates ranging from 4400 to 5900 s^−1^. To validate the experimental data, a speckle pattern was applied to the specimen surface. A high-speed camera recorded the dynamic deformation, and digital image correlation (DIC) was used to directly obtain displacement and strain for comparison with the experimental analysis. Using the DIC method, the relative motion of the region of interest (ROI) could be tracked by comparing the gray-level distributions of the reference and deformed images. An accurate strain and cracking process for concrete could thus be determined using the DIC software (MatchID 2019). An ultra-high-speed camera (Kirana-5M) with high resolution was employed to record the deformation of tensile gauge section of the M-type specimen, as shown in Figure 3. The parameters of DIC were set as shown in Table 1.

For the dynamic tensile test, a Hopkinson pressure bar with a 14.5 mm diameter was used. A copper shaper with thickness of 0.5 mm was attached to the incident bar to shape the waveform.

## 3. Numerical Simulation

To evaluate the effectiveness of the M-shaped specimen in tensile experiments, finite element FE analysis was conducted by using commercial finite element (FE) software (Abaqus/Explicit 2010). The analysis focused on wave propagation and deformation characteristics of the specimen. It aimed to verify whether stress at both ends of the specimen reached equilibrium and whether the stress distribution along the gauge length was uniform. The geometric shape of the specimen was then optimized. This optimization ensured that the M-shaped specimen met the one-dimensional stress and uniformity requirements during testing. These improvements enhanced the reliability of data processing and experimental outcomes.

### 3.1. FE Model

**Figure 4:**
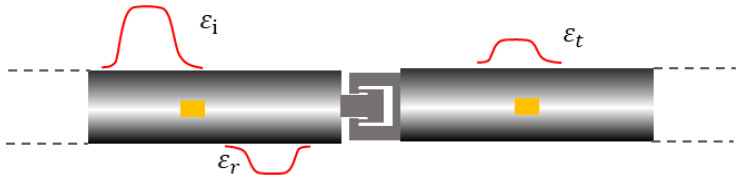
A 3D finite element model was created in Abaqus/Explicit, including the bullet, incident bar, transmission bar, and specimen.

A 3D finite element model was created in Abaqus/Explicit, including the bullet, incident bar, transmission bar, and specimen, as shown in Figure 4. The model used tetrahedral solid elements, with a minimum element size of 0.1 mm in the tensile gauge section. A simplified Johnson–Cook constitutive model, which neglected the strain rate effect of the material, was applied as follows:

(16) $$\sigma =A+B\varepsilon^{n}$$

where *A*, *B*, and *n* represent yield strength, strain hardening coefficient, and strain hardening exponent, respectively. *A* = 620 MPa, *B* = 200 MPa, *n* = 0.3, and the elastic modulus *Es* = 210 GPa.

### 3.2. Specimen Shape

**Figure 5:**
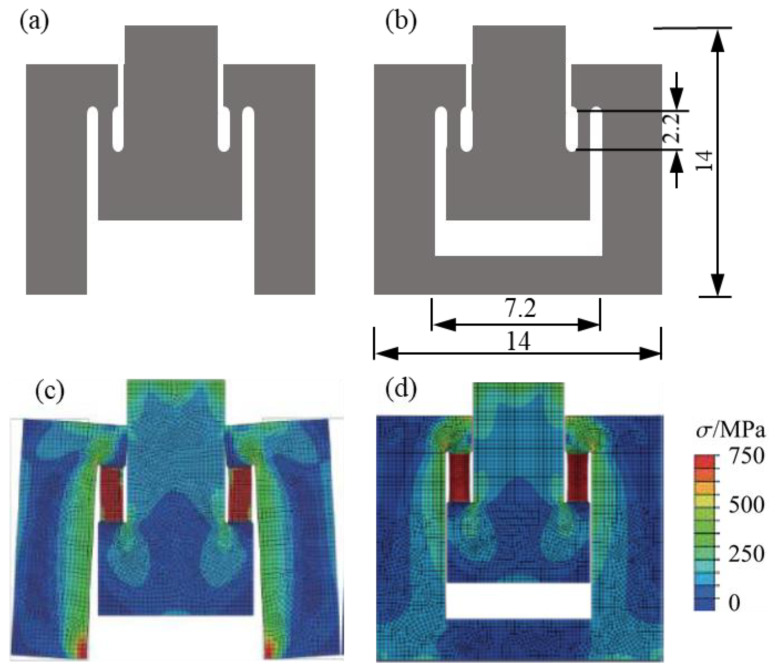
The comparation of stress and deformation for opening M-type specimen and closed M-type specimen using FE simulation: (**a**) shape of opening M-type specimen, (**b**) shape of closed M-type specimen, (**c**) the stress distribution and deformation of opening M-type specimen, and (**d**) the stress distribution and deformation of closed M-type specimen.

Mohr and Gary [29] designed an M-shaped tensile specimen with an open bottom, as shown in Figure 5a. However, FE simulations showed that the specimen’s legs opened too much, causing additional bending at the shoulder. This bending led to uneven stress distribution along the tensile gauge, as illustrated in Figure 5c.

To address this issue, it is important to reduce the effect of distortion by optimizing the specimen design. In this study, a closed M-shaped specimen, shown in Figure 5b, is proposed to increase rigidity and reduce distortion. FE simulations confirmed a more even stress distribution with this design, as seen in Figure 5d. Compared with the original state of deformation (Figure 5b), the closed M-shaped specimen was significantly improved in terms of overall bending distortion. Evidently, with no change to the main dimensions, the improved design of the closed M-shaped specimen led to a significant enhancement of overall rigidity. Also, the impact of distortion was noticeably reduced.

For a Hopkinson pressure bar with a diameter of 14.5mm, the dimensions of the specimen are detailed in Figure 5b. For the tensile gauge section, the length L_0_ was 2.2 mm, and the rectangular cross-section was 1 mm (thickness T_0_) × 1.5 mm (width W_0_).

### 3.3. Verification of Stress Balance and Uniform

For the SHPB test, the specimen is generally required to meet the one-dimensional stress assumption: “*ε_i_*(*t*) + *ε_r_*(*t*) = *ε_t_*(*t*)”. Here, FE simulations were used to evaluate the response of the M-shaped specimen and the dynamic force balance at both ends.

**Figure 6:**
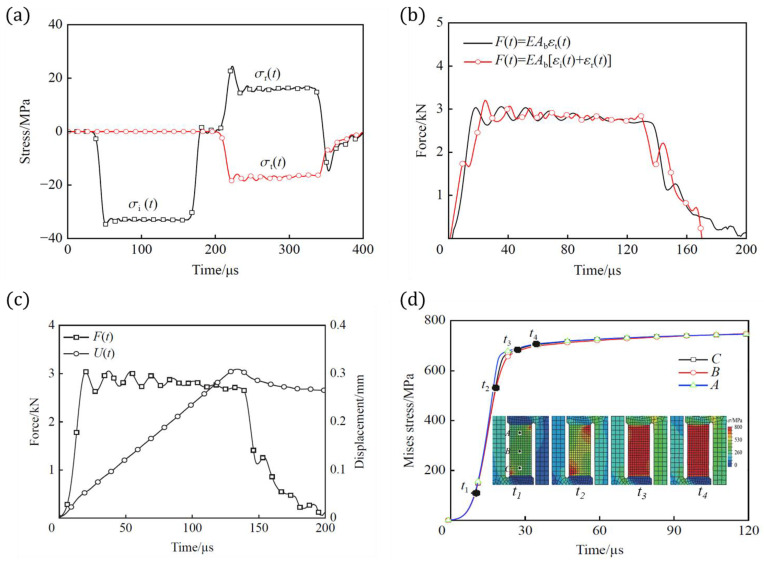
Evaluation of the response of the M-shaped specimen and the dynamic force balance at both ends: (**a**) the incident, reflected, and transmitted waves, (**b**) the force balance reached in SHPB test, (**c**) the evolution of force and deformation of specimen, and (**d**) the stress distribution was uniformed along the tensile gauge.

For the impact velocity of 2.38 m/s, the incident, reflected, and transmitted waves in the incident and transmission bars are shown in Figure 6a. Using one-dimensional stress assumption, the loading force at both ends was determined, as seen in Figure 6b. The results showed good agreement. Similar outcomes were observed for other velocities, confirming that the M-shaped specimen met the one-dimensional stress assumption and could be analyzed using Equations (13)–(15) for dynamic tensile tests.

Furthermore, the stress distribution in the tensile gauge section of the specimen was analyzed for uniformity. Figure 6c shows the force interaction between the M-shaped specimen and the transmission bar, and the compressive displacement at both ends. Figure 6d presents the stress evolution and equivalent stress–time curves at different points in the gauge section. The results showed that bending due to distortion occurred in the early loading stage (t < 27 μs, compressive displacement < 0.015 mm). As tensile deformation increased and the compressive displacement exceeded 0.035 mm, the equivalent stress at different points became uniform, and the bending effect diminished. Therefore, the tensile section deformation could be considered a one-dimensional tensile stress state.

### 3.4. Numerical Correction of Stress–Strain Curve

**Figure 7:**
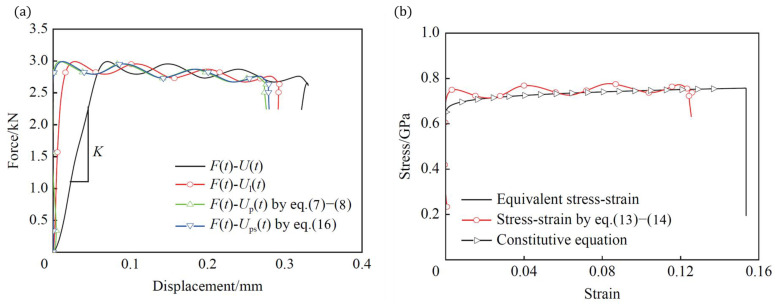
The correction of mechanical response for the M-shaped specimen: (**a**) load–displacement curve of *F*(*t*)–*U*(*t*), (**b**) the plastic true stress–strain curve.

The load–displacement curve of *F*(*t*)–*U*(*t*) for the M-shaped specimen is shown in Figure 7a. As described in Section 2.2.3, the measured displacement *U* at the specimen ends included both the deformation of the tensile section and the elastic deformation of other parts (Equation (7)). The finite element method could be used to directly obtain the actual displacement (*U_l_*) at both ends of the tensile gauge section, as shown in Figure 7a. Comparing the total displacement (*U*), we found that *U* was significantly greater than *U_l_*. Therefore, when calculating the strain in the tensile section, *U* had to be corrected to eliminate the displacement caused by the elastic deformation of the M-shaped specimen structure.

As in the simulated result, the slope of the elastic section, *K*, could be obtained, and represented the stiffness coefficient of the specimen in Equation (8). Here, for the closed M-type specimen with dimensions described in Figure 5b, it was found that *K* = 49.101 kN/mm. Then, the plastic deformation displacement *Up*(*t*) of tensile gauge section could be calculated using Equation (8).

To verify the reliability of the *Up*(*t*), *Up*(*t*) was compared with the actual plastic deformation *Ups*(*t*) of tensile gauge section. Since the tensile gauge section underwent uniaxial deformation, the displacement due to plastic deformation is given as follows:

(17) $$U_{Ps}\left(t\right)=U_{l}\left(t\right)-U_{el}\left(t\right)$$

(18) $$U_{el}\left(t\right)=\frac{F\left(t\right)L_{0}}{E_{s}A_{s}}$$

Figure 7a shows the *F*(*t*)–*Up*(*t*) curve matched well with the *F*(*t*)–*Ups*(*t*) curve, which means that the correction for deformation, as in Equation (7), was effective.

After effective data correction, the true plastic stress–strain curve for the tensile gauge section was calculated using Equations (13) and (14), as shown in Figure 7b. This plastic true stress–strain curve matched well with both the equivalent stress–strain curve of the element at the mid-point of the tensile section, and the constitutive equation curve of the material. This indicates that the plastic stress–strain curve from the Hopkinson bar dynamic tensile test accurately reflected the material’s dynamic tensile properties.

## 4. Experimental Results

### 4.1. Dynamic Test

**Figure 8:**
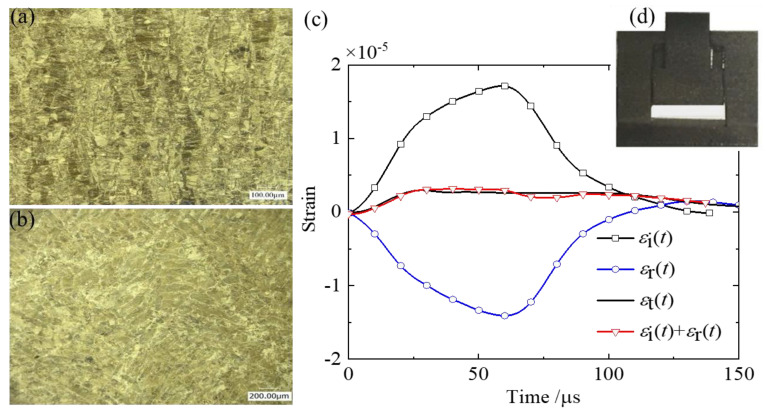
The 3D-printed stainless steel specimen and the typical stress waves measured by SHPB: (**a**) the microstructure at the direction of cross-section; (**b**) the microstructure at the direction of longitudinal section; (**c**) the typical set of incident, reflected, and transmitted wave signals obtained by SHPB; and (**d**) the 3D-printed M-type specimen.

The optimized design and validation of M-shaped tensile specimens were conducted using finite element analysis, as described in Section 3. Following the specified dimensions in Figure 5b, closed M-shaped specimens were manufactured with a 3D SLM printer. The microstructure of the cross-sectional and longitudinal sections of the tensile gauge section are shown in Figure 8a,b. The printed M-shaped specimen is displayed in Figure 8d.

A series of dynamic tensile tests was conducted on the M-shaped specimens of SLM 17-4PH stainless steel using SHPB bar. Figure 8c illustrates a typical set of incident, reflected, and transmitted wave signals. This figure also indicates the waveform of $\varepsilon_{i}\left(t\right)+\varepsilon_{r}\left(t\right)$, which is consistent with the transmitted wave $\varepsilon_{t}\left(t\right)$. It implies that the M-shaped specimens met the one-dimensional stress assumption of the SHPB test in the experiment.

### 4.2. Evolution of Tensile Stress and Strain

**Figure 9:**
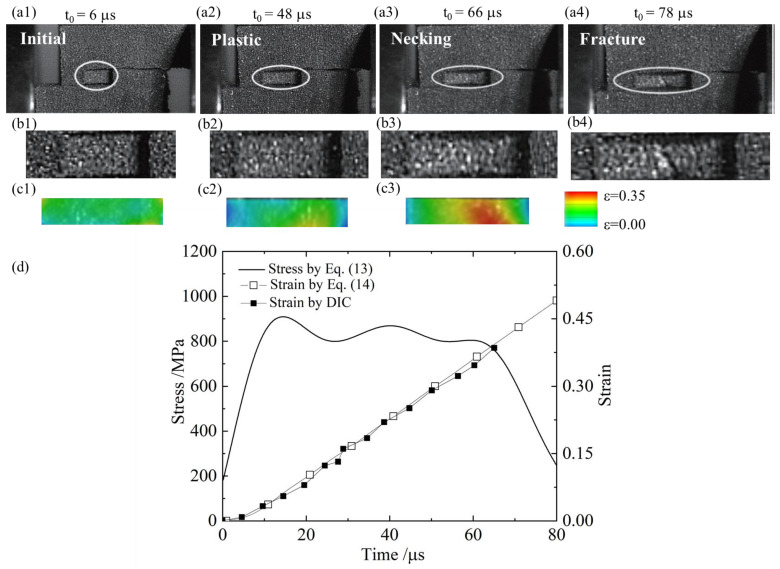
The deformation and fracture sequence of the M-type specimen (**a1**–**a4**), the detail of tensile gauge section (**b1**–**b4**), and the evolution of tensile strain analyzed by DIC method (**c1**–**c3**). The corresponding stress and strain development are shown in (**d**).

Figure 9a1–a4 capture the deformation and fracture sequence of the M-type specimen and the tensile gauge section (b1–b4). The evolution of strain at the tensile gauge section was calculated by the DIC method, as shown in Figure 9c1–c3. It is evident that, although the specimen exhibited slight distortion overall, the elongation process in the gauge section proceeded from initial uniform deformation and transitioned to necking at t = 66 μs, ultimately leading to fracture, which occurred in the middle of the gauge section. This sequence suggests that the gauge section maintained a uniform tensile state, with minimal influence from distortion-induced bending moments.

Figure 9 also shows the axial strain at the midpoint of the gauge section, measured using DIC analysis. The results demonstrate a strong correlation between the plastic strain calculated as Equation (14) and the strain measured directly by DIC, as shown in Figure 9d. This confirms the validity of the elastic correction method. Additionally, the M-type specimen met the requirements of the one-dimensional stress assumption in SHPB experiments. Therefore, Equation (14) could be used to calculate the plastic strain in the gauge section of the specimen.

### 4.3. Strain Rate

**Figure 10:**
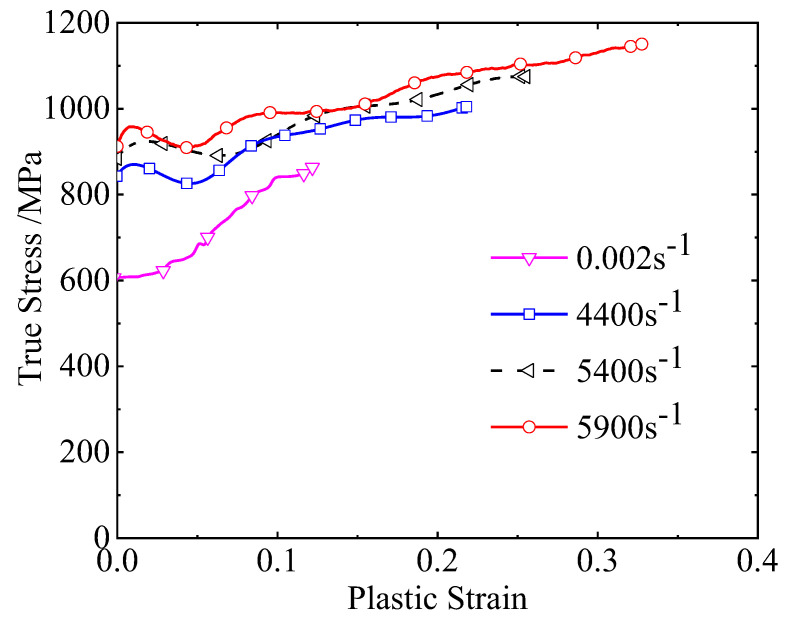
True stress–plastic strain curves of 3D-printed stainless steel obtained by M-type specimen method with different strain rates.

Using the new tensile method described in this study, M-type specimens were tested to obtain tensile stress–strain curves at strain rates ranging from quasi-static to 5900 s^−1^, as shown in Figure 10. At a strain rate of 0.002 s^−1^, 3D-printed stainless steel showed significant strain hardening, with a substantial increase in flow stress as strain increased. At strain rates between 4400 and 5900 s^−1^, the flow stress initially decreased and then increased during early plastic deformation, demonstrating a strong strain-rate hardening effect. Specifically, at 4400 s^−1^, the yield strength reached 850 MPa, and it rose to approximately 950 MPa at a strain rate of 5900 s^−1^.

The dynamic tensile test on M-type specimens using the Hopkinson pressure bar provided an effective approach to evaluating material behavior at high loading rates. Compared with the conventional split Hopkinson tensile bar, this method achieved both ultra-high strain rates (up to 6000 s^−1^) in this study, and large plastic deformations. Thus, it can serve as a reliable method for assessing the dynamic tensile properties of 3D-printed stainless steel at high loading rates.

## 5. Further Research Perspectives

Though this study provided a tensile method to investigate the dynamic tensile mechanical behaviors of 3D-printed stainless steel, this method has a limitation caused by the specimen fabrication technologies. As known, 3D-printed technology has the characteristics of internal structure and surface irregularities. These defects may alter the mechanical performance of the tested objects and distort the material’s characterization results. In fact, the stiffness of the “K” value was affected by the forming process of the 3D-printed specimen. To obtain the actual value of “K”, a direct experimental measurement was conducted by the quasi-static test [40,41,42]. The M-type specimen was compressed by an MTS universal machine with the load velocity of 0.04mm/s. The experimental result showed the “K” value was 40.6 kN/mm which was very closed to the value of 49.101 kN/mm obtained through FE analysis. The experimental results were smaller than the finite element results, which may have been related to the elastic deformation of the frame and fixture of the testing machine. Moreover, the forming process of the 3D-printed specimen may have been resulted a small discreteness in the experimental responses. Therefore, the actual “K” value will be further examined through experimental methods by improving the forming process of materials. For example, precision five-axis machining will be an alternative specimen fabrication technology suitable for the proposed dynamic tensile test.

## 6. Conclusions

This study provides a dynamic tensile method using a closed M-type specimen loaded by the SHPB. FE simulations and experiments were conducted to validate the modification of the M-type specimen manufactured by 3D SLM printer using 17-4PH stainless steel powder. These designations ensured the force balance and uniform deformation in the tensile region. The results were as follows:

1. The Hopkinson pressure bar allowed dynamic loading of closed M-type specimens while meeting the one-dimensional stress assumption. This approach eliminated connection challenges between specimens and bar ends, providing convenient loading and effective results.
2. The FE method was conducted to obtain the stiffness of M-type specimen, which was used to improve the interpretation of physical test results. This approach allowed for accurate calculation of plastic strain in the gauge section.
3. Compared with the conventional split Hopkinson tensile bar, in this study, the tensile method using the M-type specimen achieved both ultra-high strain rates (up to 6000 s^−1^) and large plastic deformations. Thus, this is a reliable method for assessing the dynamic tensile properties of stainless steel samples at high loading rates.

## Figures and Tables

**Table 1:** The DIC parameters of the analysis windows in this study.

Parameters	Setting
Subset	15 × 15 pixel^2^
Step	5 pixels
Magnification factor	0.6 mm/pixel
Strain filter size	Gaussian (5)
Matching criterion	Normalized squared differences
Interpolation	Optimized 8-tap interpolation
Shape function	Affine

## Data Availability

The original contributions presented in this study are included in the article. Further inquiries can be directed to the corresponding authors.

## References

[1] Nicholas T. (1981). Tensile testing of materials at high rates of strain: An experimental technique is developed for testing materials at strain rates up to 10^3^ s^−1^ in tension using a modification of the Split Hopkinson Bar or Kolsky Apparatus. Exp. Mech..

[2] Fu Y., Yu X., Dong X., Zhou F., Ning J., Li P., Zheng Y. (2020). Investigating the failure behaviors of RC beams without stirrups under impact loading. Int. J. Impact Eng..

[3] Liang H., Fang X., Yu X., Fu Y., Zhou G. (2023). Investigating the Fracture Process and Tensile Mechanical Behaviours of Brittle Materials under Concentrated and Distributed Boundary Conditions. Appl. Sci..

[4] Yu X., Fu Y., Dong X., Zhou F., Ning J. (2020). An Improved Lagrangian-Inverse Method for Evaluating the Dynamic Constitutive Parameters of Concrete. Materials.

[5] Weckert S.A., Resnyansky A.D. (2022). Experiments and modelling for characterisation and validation of a two-phase constitutive model for describing sands under explosive loading. Int. J. Impact Eng..

[6] Cisse C., Zaki W., Zineb T.B. (2016). A review of constitutive models and modeling techniques for shape memory alloys. Int. J. Plast..

[7] Mott P.H., Twigg J.N., Roland D.F., Schrader H.S., Pathak J.A., Roland C.M. (2007). High-speed tensile test instrument. Rev. Sci. Instrum..

[8] Zhou J., Pellegrino A., Heisserer U., Duke P.W., Curtis P.T., Morton J., Petrinic N., Tagarielli V.L. (2019). A new technique for tensile testing of engineering materials and composites at high strain rates. Proc. R. Soc. A Math. Phys. Eng. Sci..

[9] Choi I., Lee S., Matlock D.K., Speer J.G. (2006). Strain control during high speed tensile testing. J. Test. Eval..

[10] Harding J.W.E.O.C., Wood E.O., Campbell J.D. (1960). Tensile testing of materials at impact rates of strain. J. Mech. Eng. Sci..

[11] Bhujangrao T., Froustey C., Iriondo E., Veiga F., Darnis P., Mata F.G. (2020). Review of Intermediate Strain Rate Testing Devices. Metals.

[12] Hsu T., Zhang L., Gomez T. (1995). A servo-control system for the universal panel tester. J. Test. Eval..

[13] Hudson J.A., Crouch S.L., Fairhurst C. (1972). Soft, stiff and servo-controlled testing machines: A review with reference to rock failure. Eng. Geol..

[14] Xiao X. (2007). Dynamic tensile testing of plastic materials. Polym. Test..

[15] Bastias P.C., Kulkarni S.M., Kim K.Y., Gargas J. (1996). Noncontacting strain measurements during tensile tests. Exp. Mech..

[16] Roland C.M., Twigg J.N., Vu Y., Mott P.H. (2007). High Strain Rate Mechanical Behavior of Polyurea. Polymer.

[17] Wu H., Ma G., Xia Y. (2004). Experimental study of tensile properties of PMMA at intermediate strain rate. Mater. Lett..

[18] Wang W., Ma Y., Yang M., Jiang P., Yuan F., Wu X. (2018). Strain Rate Effect on Tensile Behavior for a High Specific Strength Steel: From Quasi-Static to Intermediate Strain Rates. Metals.

[19] Zhao H. (2003). Material behaviour characterisation using SHPB techniques, tests and simulations. Comput. Struct..

[20] Meng H., Li Q.M. (2003). Correlation between the accuracy of a SHPB test and the stress uniformity based on numerical experiments. Int. J. Impact Eng..

[21] Ni P., Tang L., Xu P., Wang X., Yang B., Liu Y., Liu Z., Jiang Z., Zhou L. (2025). Correction method and verification of radial inertia and friction effects under a unified deformation framework in SHPB experiments on soft materials. Int. J. Impact Eng..

[22] Sasso M., Mancini E., Chiappini G., Utzeri M., Amodio D. (2024). A 90-meter Split Hopkinson Tension–Torsion Bar: Design, Construction and First Tests. J. Dyn. Behav. Mater..

[23] Chen W.W., Song B. (2010). Split Hopkinson (Kolsky) Bar: Design, Testing and Applications.

[24] Xu Y., Lopez M.A., Zhou J., Farbaniec L., Patsias S., Macdougall D., Reed J., Petrinic N., Eakins D., Siviour C. (2022). Experimental analysis of the multiaxial failure stress locus of commercially pure titanium at low and high rates of strain. Int. J. Impact Eng..

[25] Mochalova V., Utkin A., Savinykh A., Garkushin G. (2021). Pulse compression and tension of Kevlar/epoxy composite under shock wave action. Comp. Struct..

[26] Qi L., Li H., Jin K., Feng Y., Guo Y., Li Y. (2023). Biaxial tensile behavior of Ti-6Al-4V under proportional loadings at high strain rates. Int. J. Impact Eng..

[27] Moreira B.S., Nunes P.D.P., da Silva C.M., Tenreiro A.F.G., Lopes A.M., Carbas R.J.C., Marques E.A.S., Parente M.P.L., da Silva L.F.M. (2023). Numerical Design of a Thread-Optimized Gripping System for Lap Joint Testing in a Split Hopkinson Apparatus. Sensors.

[28] Wang L.L. (2007). Foundation of Stress Waves.

[29] Mohr D., Gary G. (2007). M-shaped specimen for the high-strain rate tensile testing using a Split Hopkinson Pressure Bar apparatus. Exp. Mech..

[30] Rafi H.K., Pal D., Patil N., Starr T.L., Stucker B.E. (2014). Microstructure and Mechanical Behavior of 17-4 Precipitation Harden-able Steel Processed by Selective Laser Melting. J. Mater. Eng. Perform..

[31] Luecke W.E., Slotwinski J.A. (2014). Mechanical properties of austenitic stainless steel made by additive manufacturing. J. Res. Natl. Inst. Stand. Technol..

[32] Lu S.L., Tang H.P., Ning Y.P., Liu N., Stjohn D.H., Qian M. (2015). Microstructure and mechanical properties of long Ti-6Al-4V rods additively manufactured by selective electron beam melting out of a deep powder bed and the effect of subsequent hot isostatic pressing. Metall. Mater. Trans. A.

[33] Rodriguez O.L., Allison P.G., Whittington W.R., Francis D.K., Rivera O.G., Chou K., Gong X., Butler T.M., Burroughs J.F. (2015). Dynamic tensile behavior of electron beams additive manufactured Ti6Al4V. Mater. Sci. Eng. A.

[34] Mcwilliams B., Pramanik B., Kudzal A., Taggart-Scarff J. (2018). High strain rate compressive deformation behavior of an additively manufactured stainless steel. Addit. Manuf..

[35] Shi T.Y., Liu D.S., Chen W., Xie P.C., Wang X.F., Wang Y.G. (2019). Dynamic tensile behavior and spall fracture of GP1 stainless steel processed by selective laser melting. Explos. Shock Waves.

[36] Song B., Nishida E., Sanborn B., Maguire M., Adams D., Carroll J., Wise J., Reedlunn B., Bishop J., Palmer T. (2017). Compressive and tensile stress–strain responses of additively manufactured (AM) 304L stainless steel at high strain rates. J. Dyn. Behav. Mater..

[37] Wang X., Wang G., Shi T., Wang Y. (2021). Tensile mechanical behavior and spall response of a selective laser melted 17-4 PH stainless steel. Metall. Mater. Trans. A.

[38] Ding L., Li H.X., Wang Y.D., Huang Z.T. (2015). Heat Treatment on Microstructure and Tensile Strength of 316 Stainless Steel by Selective Laser Melting. Chin. J. Lasers.

[39] Lee W., Lin C. (1998). Plastic deformation and fracture behaviour of Ti–6Al–4V alloy loaded with high strain rate under various temperatures. Mater. Sci. Eng. A.

[40] Cestino E., Frulla G., Piana P., Duella R. (2020). Numerical/Experimental Validation of Thin-Walled Composite Box Beam Optimal Design. Aerospace.

[41] Gürdal Z., Tatting B.F., Wu C.K. (2008). Variable stiffness composite panels: Effects of stiffness variation on the in-plane and buckling response. Compos. Part A Appl. Sci. Manuf..

[42] Pierron F., Vert G., Burguete R., Avril S., Rotinat R., Wisnom M.R. (2007). Identification of the orthotropic elastic stiffnesses of composites with the virtual fields method: Sensitivity study and experimental validation. Strain.

